# A63 RESTRAINED STRESS IN MICE INDUCES TRANSLOCATION OF GUT COMMENSAL BACTERIAL INTO DISTAL ORGANS

**DOI:** 10.1093/jcag/gwad061.063

**Published:** 2024-02-14

**Authors:** C Shimbori, N Kraimi, J Lu, G De Palma, S Collins, P Bercik

**Affiliations:** Medicine, McMaster University, Hamilton, ON, Canada; Medicine, McMaster University, Hamilton, ON, Canada; Medicine, McMaster University, Hamilton, ON, Canada; Medicine, McMaster University, Hamilton, ON, Canada; Medicine, McMaster University, Hamilton, ON, Canada; Medicine, McMaster University, Hamilton, ON, Canada

## Abstract

**Background:**

Bacterial translocation is defined as the migration of bacteria from the intestinal lumen into extraintestinal tissues; this process has been implicated in the pathophysiology of gastrointestinal disorders including irritable bowel syndrome (IBS) and central nervous system disorders. In our recent study, we found that gut bacteria from patients with IBS can disrupt the mucus layer and penetrate into the *lamina propria* in our microbiota-humanized mouse model. It is well known that psychological stress triggers or exacerbates IBS symptoms. Furthermore, experimental stress models have been able to reproduce some features of IBS and demonstrate that gut bacteria can translocate into the mesenteric lymph node (MLN), liver, and spleen. These studies suggested that bacterial translocation might induce changes in behavior and brain function. However, the direct causal relationship between bacterial translocation and behavioral changes, and the mechanism of bacterial translocation remains unclear.

**Aims:**

To investigate translocation of commensal bacteria within the gut wall and their migration into distant organs using a mouse stress model.

**Methods:**

SPF C57BL/6 mice were split into 2 groups: restraint stress group (3 hrs x 7 days, n=9) and non-stress control group (n=8). After the last session of stress, we conducted light/dark preference and tail suspension tests. We collected jejunum, ileum, and colon samples for histology. We also sampled blood, MLN, liver, spleen, and brain for histology and bacterial cultures. The tissues were collected under aseptic conditions. The blood or organ homogenates were plated and incubated in aerobic and anaerobic conditions. Intestinal tissues were submitted to fluorescence in situ hybridization staining with a bacterial universal probe in combination with immunofluorescence to co-stain with e-cadherin and CD103. The images were taken by confocal microscopy.

**Results:**

The chronic restrain stress-induced anxiety-like, but not depressive behavior, as assessed by the light/dark test. Compared to the controls, bacterial culture was more frequently positive in the stress group: blood (0% vs 25%), MLN (0% vs 50%), spleen (0% vs 25%), liver (0% vs 25%), and brain (25% vs 74%). The histology showed that intestinal bacteria were present within the gut wall and brain in the stress group. Interestingly, these bacteria co-localized with the goblet cells in the jejunum, ileum and colon, and CD103^+^ dendritic cells in the colon (Fig. 1).

**Conclusions:**

We found that mice subjected to the restraint stress exhibit abnormal behavior, accompanied by bacterial infiltration into the gut wall, as well as bacterial translocation into distal organs, including brain. Our histology data suggest a potential contribution of goblet cells-dendritic cells pathway in commensal gut bacteria translocation in the restrained stress model.

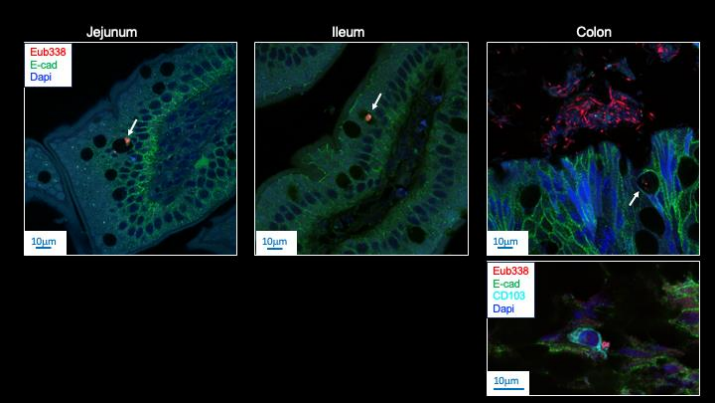

Intestinal bacteria were present within the gut wall in the stress group.

**Funding Agencies:**

CIHR Farncombe Innovation Fund

